# Identification and Characterization of Multiple Intermediate Alleles of the Key Genes Regulating Brassinosteroid Biosynthesis Pathways

**DOI:** 10.3389/fpls.2016.01893

**Published:** 2017-01-16

**Authors:** Junbo Du, Baolin Zhao, Xin Sun, Mengyuan Sun, Dongzhi Zhang, Shasha Zhang, Wenyu Yang

**Affiliations:** ^1^College of Agronomy, Sichuan Agricultural University, Sichuan Engineering Research Center for Crop Strip Intercropping SystemChengdu, China; ^2^Ministry of Education Key Laboratory of Cell Activities and Stress Adaptations, School of Life Sciences, Lanzhou UniversityLanzhou, China; ^3^Key Laboratory of Tropical Plant Resources and Sustainable Use, Xishuangbanna Tropical Botanical Garden, Chinese Academy of ScienceKunming, China

**Keywords:** brassinosteroid, brassinolide, intermediate alleles, BR biosynthesis, BR catabolism

## Abstract

Most of the early identified brassinosteroid signaling and biosynthetic mutants are null mutants, exhibiting extremely dwarfed phenotypes and male sterility. These null mutants are usually unable to be directly transformed via a routinely used *Agrobacterium*-mediated gene transformation system and therefore are less useful for genetic characterization of the brassinosteroid (BR)-related pathways. Identification of intermediate signaling mutants such as *bri1–5* and *bri1–9* has contributed drastically to the elucidation of BR signaling pathway using both genetic and biochemical approaches. However, intermediate mutants of key genes regulating BR biosynthesis have seldom been reported. Here we report identification of several intermediate BR biosynthesis mutants mainly resulted from leaky transcriptions due to the insertions of T-DNAs in the introns. These mutants are semi-dwarfed and fertile and capable to be transformed. These intermediate mutants could be useful tools for future discovery and analyses of novel components regulating BR biosynthesis and catabolism via genetic modifier screen.

## Introduction

Brassinosteroids (BRs) are a group of plant steroidal hormones playing vital roles in almost all aspects of plant growth and development. Mutants blocking either BR signaling transduction or biosynthesis exhibit typical phenotypes including dwarfism, de-etiolation in darkness, delayed flowering, reduced male fertility, and short root phenotypes (Clouse and Sasse, 1998). Since the BR receptor BRASSINOSTEROID-INSENSITIVE 1 (BRI1) was discovered along with a series of *bri1* mutants obtained by mutagenesis, multiple typical weak alleles of *bri1* showing semi-dwarf phenotypes have been characterized (Li and Chory, 1997; Noguchi et al., 1999). For example, *bri1–5* is one of the first described and wildly used weak BR mutants in WS2 ecotype which bears a substitution of Tyr for Cys at the 69th amino acid (aa) of the paired cysteines in the extracellular domain (Noguchi et al., 1999). In addition, *bri1–6, bri1–7, bri1–8*, and *bri1–9* are the other ethyl methane sulfonate (EMS)-mutagenic *bri1* mutants in WS2 ecotype with different amino acid substitution (Noguchi et al., 1999). *bri1–6* (G644D) and *bri1–7* (G613S) are two mutants mutated in the 70 aa island in the extracellular domain. A change of Arg to Asn at 983th aa in the kinase domain in *bri1–8* was identified. *bri1–9* carries a missense mutation of Ser622-to-Phe in the 21st LRR of the extracellular domain. Further investigation showed that *bri1–5* and *bri1–9* were retained in the endoplasmic reticulum (ER) by an ER quality control system (Jin et al., 2007; Hong et al., 2008). *bri1–119* is a mutant with G644D in En-2 ecotype by point mutagenesis (Friedrichsen et al., 2000). A Gly was replaced by Ile at the 989th aa of the kinase domain in *bri1–301* of Col-0 ecotype (Xu et al., 2008). *bri1–120* has a change of Ser to Phe at the 399th aa in the 13th LRR domain of BRI1 (Shang et al., 2011).

During the past decades, the intermediate alleles of *bri1* in Arabidopsis have helped with identification of new components involving in BR signaling and biosynthesis pathways. For instance, BRI1 SUPPRESSOR 1 (BRS1), BRI1-ASSOCIATED KINASE 1 (BAK1), BRI1-LIKE RECEPTOR KINASE 1 (BRL1), and BSU1 are discovered to be involved in BR signal transduction by genetic suppressor screen for *bri1–5* (Li et al., 2001, 2002; Mora-García et al., 2004; Zhou et al., 2004). *bri1-EMS-suppressor 1* (*bes1-D*) was identified as a suppressor of *bri1–119* (Yin et al., 2002). *activation-tagged bri1 suppressor-Dominant* (*atbs-D*) can suppress the dwarf phenotype of *bri1–301* (Wang et al., 2009; Kang et al., 2010). *EMS-mutagenized bri1 suppressor 2* (*ebs2*) is an allele-specific suppressor of *bri1–9*. TCP1 was found as a transcription factor of *DWARF4* (*DWF4*), which encodes a rate-limiting enzyme in BR biosynthetic pathway, by suppressor screen for *bri1–5* (Guo et al., 2010). *bri1–5* ENHANCED 1 (BEN1) is an enhancer of *bri1–5*, which is responsible for BR metabolism and whose overexpression enhanced the dwarf phenotype of *bri1–5* (Yuan et al., 2007).

BR biosynthetic pathways were annotated largely by using gas chromatography-mass spectrometry (GC-MS) and genetic modifier screening in recent years. To date, several key enzymes such as DE-ETIOLATED2 (DET2), DWF4, CONSTITUTIVE PHOTOMORPHOGENESIS AND DWARFISM (CPD), CYP90C1/ ROTUNDIFOLIA (ROT3), CYP90D1, BR6OX2/CYP85A2 have been found to catalyze the conversion of the intermediates in the BR biosynthesis pathways in Arabidopsis (Fujioka et al., 1997; Choe et al., 1998; Kim et al., 1998, 2005; Shimada et al., 2003; Ohnishi et al., 2012). It is revealed that BR was biosynthesized via one of the sterol biosynthetic pathways from the 24-methylenelophenol. The 24-methylenelophenol can be converted into at least three final products in sterol biosynthetic pathways: Stigmasterol, brassicasterol and BRs. When the 24-methylenelophenol is catalyzed to 24-ethylidenelophenol by sterol methyltransferases SMT2 and SMT3, the intermediate 24-ethylidenelophenol is then converted to acenasterol catalyzed by C4-methyltransferase, to 5-dehydroavenasterol by Δ^7^-sterol-C5-desaturase DWF7, to isofucosterol by sterol Δ^7^ reductase DWF5, to Δ^24, 25^-sitosterol and then to sitosterol by the 24-methylenecholesterol reductase DWF1, and the sitosterol is finally converted to stigmasterol by C-22 sterol desaturases (Choe et al., 1999a,b, 2000; Clouse, 2002). When the 24-methylenelophenol is catalyzed by C4-demethylase, it converted into episterol, then to 5-dehydro eposterol catalyzed by DWF7, to 24-methyldesmosterol by DWF1 (Choe et al., 1999a,b, 2000; Clouse, 2002). 24-methyldesmosterol then can be converted to campesterol (CR) for brassinosteroid biosynthesis, or to 24-epi-campesterol for brassicasterol biosynthesis. The precursor CR is principally converted to campestanol (CN) by CPD and DET2, and then to BRs via the early and late C-6 oxidation pathways (Zhao and Li, 2012). In these processes, the C-22 hydroxylase DWF4 is a rate-limiting enzyme which can catalyze multiple C-22 hydroxylation steps. CN can be orderly converted to 6-oxocampestanol (6-oxoCN), cathasterone (CT), teasterone (TE), 3-dehydroteaserone (3DT), typhasterol (TY), and then to castasterone (CS), respectively, in the early C-6 oxidation pathway. For the late C-6 oxidation pathway, CN is converted to 6-deoxocathasterone (6-deoxoCT) and then to CS as the similar steps to those in the early C-6 oxidation pathway. The CS is then to finally catalyzed to BR (Zhao and Li, 2012).

Meanwhile, plants would evoke a precise metabolic system to maintain the intracellular BR homeostasis to ensure optimal growth and development. BR catabolic pathways are still largely unknown. Only a few components were found to participate in BR catabolic pathways largely by mutant screening so far. For example, ACTIVATION-TAGGED SUPPRESSOR 1 (BAS1) involves in BR catabolism in Arabidopsis and provides a connection between photoreceptor signal transduction and BR signaling pathways (Neff et al., 1999; Turk et al., 2003). BEN1, a dihydroflavonol 4-reductase (DFR)-like protein, is responsible for regulating the levels of TY, CS, and BL (Yuan et al., 2007). DRL1, an acyltransferase, regulates BR homeostasis likely by catalyzing the BR conjugation through esterification (Zhu et al., 2013). BRASSINOSTEROID INACTIVATOR1 (BIA1) and ABNORMAL SHOOT-1 (ABS-1), two BAHD acyltransferase family proteins, have been found to be involved in BR acylation to reduce BR levels (Roh et al., 2012; Wang et al., 2012). The acyltransferase PIZZA (PIZ) can modulate BR levels by acylation in Arabidopsis (Schneider et al., 2012).

More precise and detailed mechanisms of BR biosynthesis and catabolism need to be investigated. Limitation of intermediate BR biosynthetic mutants leads to slow progress in this research area. Thus, far, only a few weak mutants of key BR biosynthetic enzymes encoding genes have been found in Arabidopsis, such as *det2–1, det2–28, det2–101, psc1*/*dwf4* (Chory et al., 1991; Li et al., 2001; Ren et al., 2009; Guo et al., 2010). In this study, we report the identification of several intermediate BR biosynthetic mutants showing semi-dwarf phenotypes, which would be excellent materials for genetic modifier screen to discover new components in BR biosynthesis and catabolism pathways.

## Materials and methods

### Plant materials and growth condition

Seeds of Col-0, *dwf4–96* (SAIL_580_B09), *dwf4–44* (SAIL_882_F07), *cpd91* (SALK_078291), *cpd* (SALK_023532) and *dwf5–7* (SALK_127066) were ordered from Arabidopsis Biological Resource Center (ABRC). *dwf5–8* was obtained by T-DNA insertional mutagenesis in the *bak1–4* background. Briefly, a binary vector *pBIB-BASTA* was transformed into *bak1–4* mutant, a mutant showing a dwarf phenotype similar to that of BR biosynthetic mutants was obtained in the T2 transgenic plants. The dwarf mutant in the *bak1–4* background was then crossed with Col-0 or *bak1–4* for several generations to segregate out other mutation sites. Arabidopsis seeds were surface-sterilized and grown in the soil or on the 1/2 Murashige and Skoog (MS) media (pH 5.7) supplemented with 1% sucrose and 0.8% agar and then placed in a greenhouse at 22°C under 16 light/8 h dark condition.

### BR treatment and root growth assay

Surface-sterilized seeds were grown in the soil by spraying 1 μM of 24-epiBL twice a day, then plant rosette width were measured after 3 weeks. For root growth assay, surface-sterilized seeds were grown on 1/2 MS plates supplemented with 1% sucrose, 0.8% agar with different concentrations of 24-epiBL. The seeds were then stratificated at 4°C for 2 days and placed at 22°C under 16 h light/8 h dark conditions. Roots of 7-day-old seedlings were measured and analyzed using Graphpad Prism and ImageJ v1.47m software. At least ten plants were used in each measurements. All experiments were repeated for at least 3 times. Only the represented seedlings and statistical data were shown. Student's *t*-test was used to show the significance between the mutants.

### BRZ treatment and hypocotyl growth inhibition

Seeds were grown on 1/2 MS plates with different concentrations of brassinazole (Chemiclones, Canada) at 22°C under 16 light/8 h dark condition. Hypocotyls of 5-day-old seedlings were measured and analyzed using Graphpad Prism and ImageJ v1.47m software. At least ten plants were used in each measurements. All experiments were repeated for at least 3 times. Only the represented seedlings and statistical data were shown. Student's *t*-test was employed to show statistical differences between the mutants.

### Rosette width, plant height, silique length, and seed number measurements

Plants grown in the soil just before bolting were used for rosette width measurement. Matured plants grown in the soil were used for plant height, silique length, and seed number measurements. Graphpad Prism and ImageJ v1.47m software was used to analyze the data. At least ten plants were used in each measurements. All experiments were repeated for at least 3 times. Only the represented seedlings and statistical data were shown. Student's *t*-test was used to show statistical differences between the mutants.

### Semi-quantitative reverse transcription PCR (RT-PCR) and quantitative RT-PCR (qPCR) analyses

Two micrograms of total RNA extracted from seedlings were reversely transcribed by using M-MLV Reverse Transcriptase (Thermo Fisher Scientific). First strand cDNAs of reversely transcribed 50 ng of RNA was used for semi-quantitative RT-PCR analyses with *ExTaq* DNA polymerase (TaKaRa) and qPCR with Universal SYBR® GREEN qPCR Master Mix (2 ×) (Gangchi Bio). The parameters of semi-quantitative PCR is as follows: 95°C for 5 min, 95°C for 15 s, 50°C for 30 s, 72°C for 1 kb min^−1^, go to step 2 for another more cycles according to the expression level of the specific genes. Parameters of the qPCR is 95°C for 3 min, 95°C for 15 s, 55°C for 15 s, 72°C for 20 s, go to step 2 for 39 more cycles. Then increment of 0.5°C from 65°C to 95°C for 5 s was used for melt curve analysis. ΔΔCq method was used to normalize the qPCR data. *ACT2* was used as an internal control. Primers are listed in Table S1.

### Gene cloning and transformation

Total RNA was extracted from 2-week-old plants by using RNAprep pure Plant Kit (TIANGEN). Two micrograms of total RNA was transcribed by using M-MLV Reverse Transcriptase (Thermo Fisher Scientific). cDNAs were then amplified from reversely transcribed total RNA and constructed into pDONR/Zeo with BP clonase II by a Gateway® Cloning technology (Invitrogen, USA). The ENTR plasmid was then recombined into a binary vector *pBIB-BASTA-35S-GWR-GFP* with LR clonase II and transformed in the corresponding mutants via an agrobacteria-mediated transformation (Du et al., 2016).

### Western blot assay

Seven-day-old seedlings were treated with 0 and 100 nM 24-epiBL for 90 min and then grounded to fine powder in the liquid N_2_ for total protein isolation (Wang et al., 2005). Proteins were immuno-blotted with α-BES1 antibodies after being separated on 12% SDS-PAGE gel according to previous study (Gou et al., 2012).

## Results

### Identification and verification of *dwf5–8* mutant

**Figure 1:**
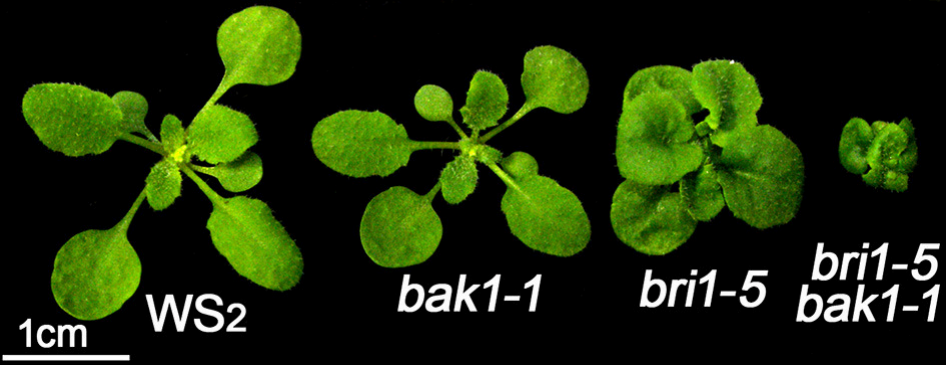
***bri1–5 bak1–1* double mutant shows a more severe dwarf phenotype than that of both *bri1–5* and *bak1–1* single mutants**. Three-week-old plants were photographed. Scale bar represents 1 cm.

**Figure 2:**
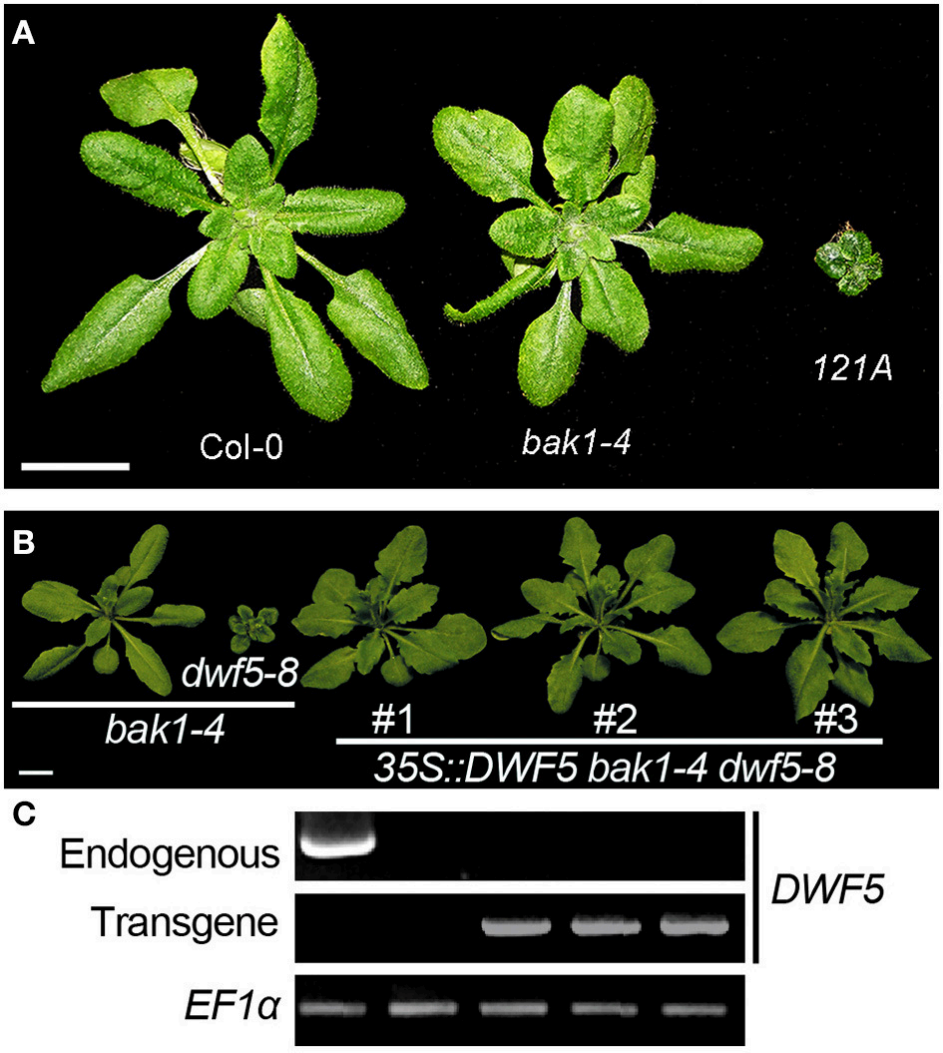
**The dwarf phenotype of *121A* was caused by the mutation of *DWF5***. **(A)** 121A shows an extremely dwarf phenotype compared to Col-0 and *bak1*−*4*. 121A was obtained from the pool 121 by a T-DNA insertional mutagenesis in the *bak1*−*4* single mutant. Scale bar represents 1 cm. **(B)** Phenotypes of the *bak1*−*4 dwf5-8* and the rescued transgenic lines. Overexpression of *DWF5* fully restored the dwarfism of *bak1*−*4 dwf5*−*8* double mutant. Scale bar represents 1 cm. **(C)** RT-PCR analyses of endogenous and the transgenes of *DWF5* (36 cycles). *EF1*α was amplified as a control for 19 cycles.

BAK1 is essential for brassinosteroid perception as a co-receptor of BRI1. The single mutant *bak1–1* shows a subtle phenotype which mimics the weak allele *bri1–5*, whereas double mutant of *bri1–5 bak1–1* exhibit more serious dwarfism than that of both the two single mutants (Figure 1). Moreover, double mutation of *BAK1* and its functionally redundant gene *BKK1* shows a spontaneous cell death symptom (He et al., 2007). Therefore, we speculate that BAK1 might have collaborated with other unknown components including BR biosynthesis or signaling pathway-related genes to regulate plant growth and development. A T-DNA mutagenesis was employed to screen mutants in *bak1–4* background with *pBIB-BASTA* via agrobacteria-mediated transformation. In T2 transgenic plants, a mutant named 121A from pool 121 showing a dwarf phenotype was obtained, which mimics BR-deficient mutants (Figure 2A). 121A was then crossed with Col-0 to segregate out the *bak1–4* background, which was named as 121ANb. RT-PCR analyses were used to detect expression of key BR biosynthesis genes. The results show that *DWF5* but not *CPD, BR6OX2, DW4, DET2, DWF1*, and *DWF7* were not detectable in the 121ANb mutant (Figure S1), suggesting that *DWF5* might be knocked out. Then we designed several primers to amplify the genomic DNA fragments to investigate whether there is a mutation in *DWF5*. The results show that the PCR products cannot be examined with primers of DWF5-5UTR F and DWF5-5UTR R, indicating that there might be some mutation in the sequence between the two primers (Figure S2). Further analyses show that a deletion of −1213 to 376 bp from the start codon of *DWF5* was found in 121ANb by PCR-based sequencing using primers of DWF5-Middle F and DWF5-Middle R (Figure S3), indicating that the dwarf phenotype might be caused by the mutation of *DWF5* or AT1G05440. The single dwarf mutant was then named after *dwf5–8*. Overexpression of *DWF5* restored the dwarfism phenotypes of *bak1–4 dwf5–8* double mutant, suggesting that the dwarf phenotype is truly caused by loss-of-function of *DWF5* (Figures 2B,C).

### The intermediate BR biosynthesis mutants show semi-dwarf phenotypes

**Figure 3:**
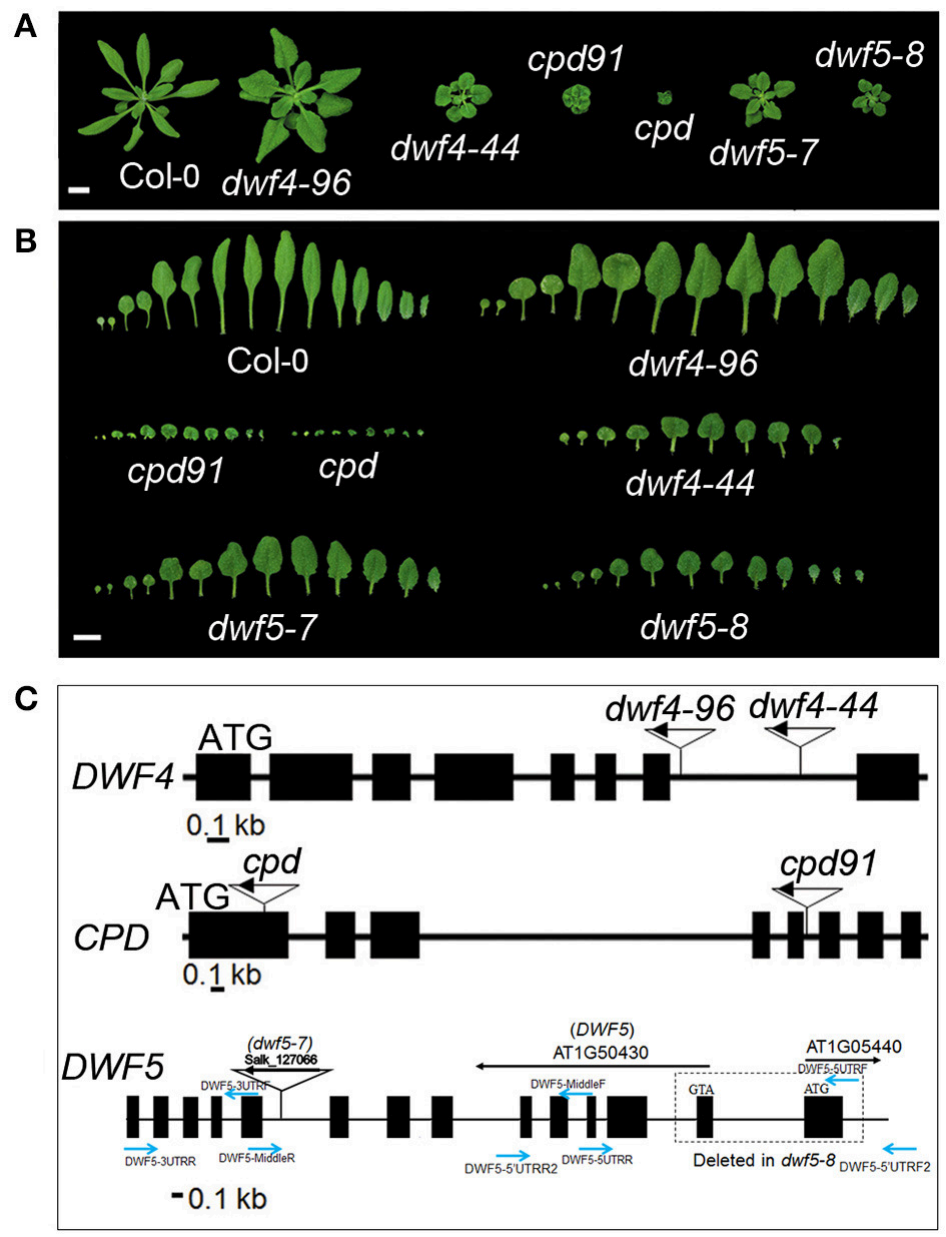
**The intermediate mutants show less compact phenotypes than that of the null mutants**. **(A)** Weak alleles of *dwf4, cpd*, and *dwf5* show less compact phenotypes than that of the null mutants. **(B)** The detached rosette leaves of the weak mutants show longer petioles and leaves than that of the null mutants. **(C)** T-DNA insertion sites in the mutants. Blue arrows show the primer sites for *dwf5*−*8* identification.

Mutant analyses were often used in researches on BR biosynthesis. For the purpose of further discovery of unknown components in BR biosynthesis pathway by genetic approaches, several biosynthetic mutants have been obtained from the ABRC (Arabidopsis Biological Resource Center) or made locally using a T-DNA insertional mutagenesis. *dwf4–96* (SAIL_580_B09) and *dwf4–44* (SAIL_882_F07) are two new mutants of *DWF4* found in the Col-0 ecotype. *dwf4–96* shows round and dark-green rosette leaves, while *dwf4–44* shows more severe dwarfism and shorter petiole phenotypes (Figures 3A,B). Both of them possess a T-DNA insertion in the seventh intron (Figure 3C). qPCR analyses show that *DWF4* was expressed in both of the *dwf4* mutants (Figure S4). *cpd91* (SALK_078291), harboring a T-DNA insertion in the fifth intron of AT5G05690 was identified as an additional mutant of *CPD* showing a bigger rosette than that of the null allele *cpd* (SALK_023532) (Figures 3A–C). *cpd91* is a leaky mutant confirmed by qPCR analysis (Figure S4).

**Figure 4:**
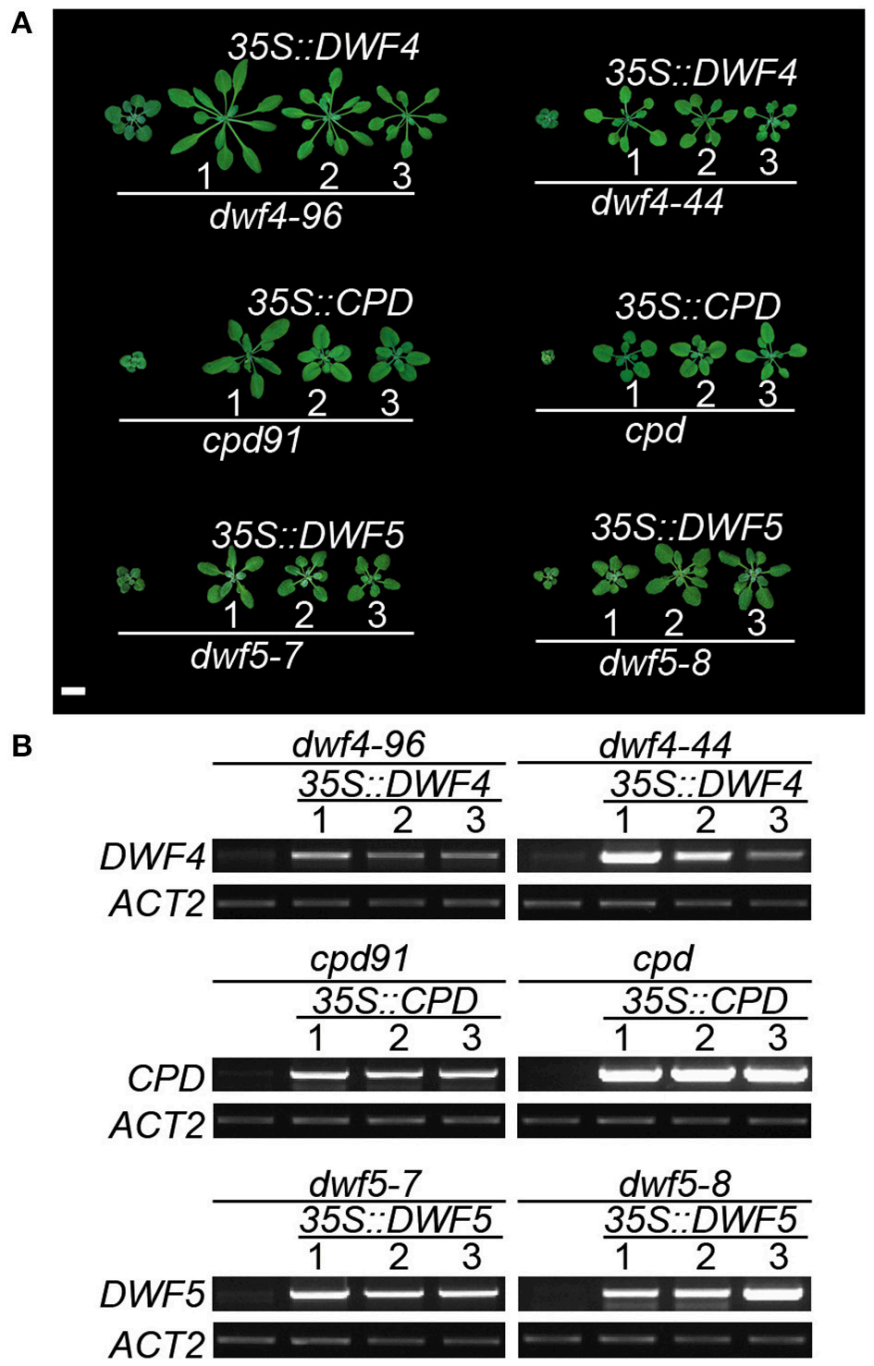
**Overexpression of genes restored the weak and null mutants**. **(A)** Phenotypes of the mutants and the rescued transgenic plants. Scale bar represents 1 cm. **(B)** RT-PCR analyses show the mutants are rescued by the overexpression of corresponding genes (30 cycles). *ACT2* was amplified as a control for 19 cycles.

Six alleles of *dwf5* showing typical dwarfism phenotypes resembling the other BR mutants have been described previously (Choe et al., 2000). In addition to *dwf5–8*, here we acquired another *dwf5* alleles, *dwf5–7* (SALK_127066) from ABRC (Figures 3A,B). *dwf5–7* is a leaky mutant obtained from ABRC harboring a T-DNA insertion in the eighth intron of *DWF5* (Figure 3C). Expression of *DWF5* is not detectable in both *dwf5–7* and *dwf5–8* mutants. Moreover, all the dwarfism and short petiole phenotypes of the mutants can be rescued by overexpression of the corresponding genes (Figures 4A,B). Relative expression of *ROT3* is higher, while *SAUR-AC1* is lower in *dwf4–96, dwf4–44, cpd91, cpd, dwf5–7*, and *dwf5–8* mutants than Col-0 (Figure S4). These results clearly suggest that the newly identified mutants are indeed caused by mutation of genes involving in BR biosynthesis pathways.

### Responses of the mutants to brassinolide (BL) and brassinazole (BRZ)

**Figure 5:**
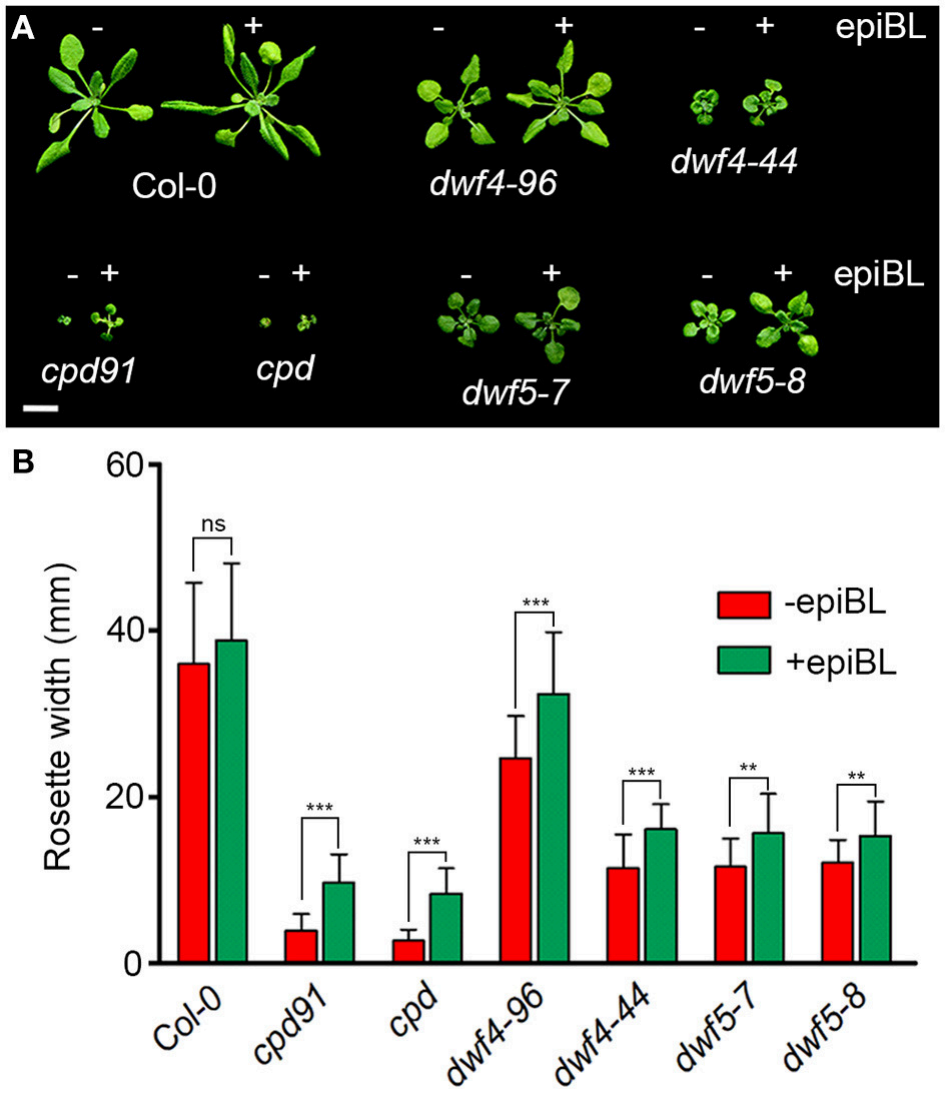
**The dwarf phenotype of *dwf4–96, dwf4–44*, *cpd91*, *cpd*, *dwf5–7*, *dwf5–8* were significantly restored by exogenous 1 μM of 24-epiBL feeding. (A)** Phenotypes of seedlings been sprayed with 0 or 1 μM of 24-epiBL twice a day for 3 weeks. Scale bar represents 1 cm. **(B)** Rosette width of 3-week-old plants with 0 or 1 μM of exogenous 24-epiBL feeding. Two-tailed *t*-test was used to show significance between the 24-epiBL-untreated and treated rosette width of the seedlings. (^***^P < 0.001; ^**^P < 0.01).

It is well known that exogenous BL feeding can rescue the dwarf phenotypes of BR biosynthetic mutants. To test whether these mutants can be truly rescued by BL feeding, soil-grown plants of Col-0 and *dwf4–96, dwf4–44, cpd91, cpd, dwf5–7*, and *dwf5–8* mutants were sprayed with 1 μM of 24-epiBL for 3 weeks, the results show that these mutants are all remarkably restored in plant size by spraying 1 μM of epiBL (Figure 5). In addition, root length of *cpd91, dwf5–7, dwf5–8* can be significantly restored by low concentration of BL treatment, and hypocotyl length of some of the mutants was elongated more or less by BL treatment (Figures S5A–C). The results indicate that these mutants exhibit responses similar to other known BR-deficient mutants to BL feeding.

One of the critical roles of BRs is promoting the hypocotyl elongation. In an attempt to understand the effects of the mutants on the hypocotyl growth, we applied the BR-specific inhibitor, BRZ on the mutants in the dark (Asami et al., 2000). The results show that 0.1 and 0.5 μM BRZ inhibited the hypocotyl length of mutants more or less (Figure S6). The *dwf4–96* and *dwf5–7* mutants show less sensitive to 0.1 μM BRZ than another set of alleles, *dwf4–44* and *dwf5–8*, respectively. However, *cpd91* shows similar response to BRZ with the null allele *cpd*, probably because these two mutants exhibit not significant difference of dwarfism at early growth stages. The application of 24-epiBL and BRZ suggests that these dwarf mutants indeed resulted from blocking BR biosynthesis in different degrees.

### The mutants differentially display biochemical sensitivity to BR response

**Figure 6:**
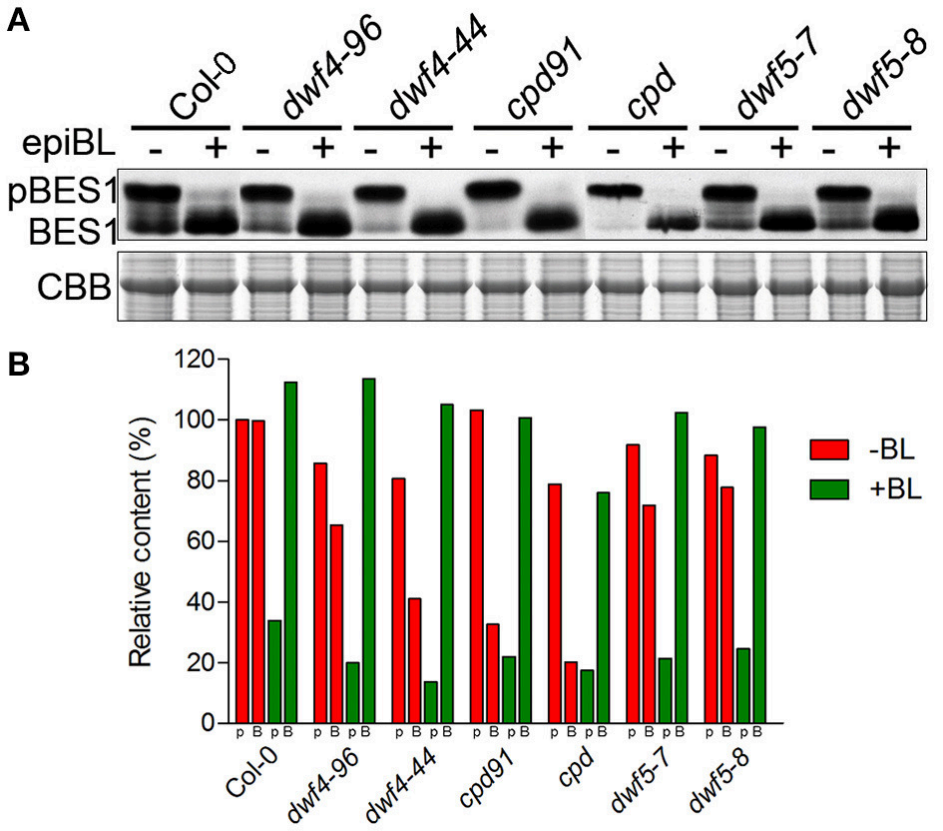
***In vivo* assay of BES1 activity shows that dephosphorylation of BES1 was impaired in the mutants and BL feeding fully restored the BR-response of the BR-deficit mutants**. **(A)** Western blotting to detect phosphorylation and dephosphorylation status of BES1. **(B)** Relative amount of phosphorylated and dephosphorylated BES1 in the mutants according to the western-blot results of **(A)**.

The dephosphorylated BES1, the active form, entering the nuclei is a key step in BR signaling pathway (Yin et al., 2002). To test whether the mutants we obtained affect the BR signaling pathway, western-blot was employed to examine the phosphorylation and the dephosphorylation status of BES1 in the mutants. The results show that the dephosphorylated BES1 was decreased in *dwf4, cpd*, and *dwf5* mutants, and the dephosphorylation was restored by application of 24-epiBL, indicating that these mutants impaired the activities of BES1 in BR signaling pathway (Figures 6A,B).

### The intermediate alleles are good tools for genetic suppressor screen

**Figure 7:**
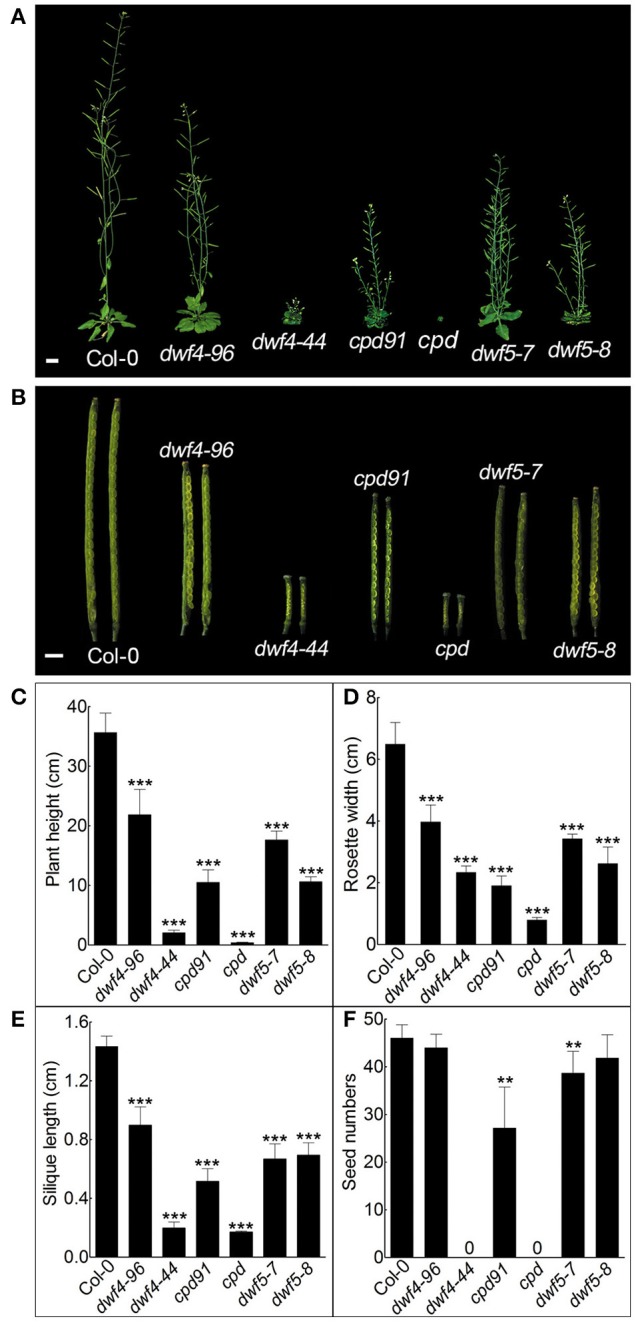
**Phenotypes of the matured null and weak allele mutants. (A)** Plant height of the matured plants. The weak mutants show semi-dwarf phenotypes. Scale bar represents 1 cm. **(B)** The peeled silique phenotypes of the null and weak allele mutants. Scale bar represents 1 mm. **(C)** Statistical analyses of plant height. **(D)** Statistical data for the rosette width of the mutants. **(E)** Silique length measurements of the mutants. **(F)** Seed number count of the mutants. One-tailed *t*-test was used to show significance of the index between Col-0 and the mutants. (^***^P < 0.001; ^**^P < 0.01).

It is proven that genetic suppressor screening is an effective approach in researches on BRs. Because a typical null BR mutant shows extreme dwarf and sterility phenotypes, the ideal mutants often chosen for genetic modifier screen are intermediate mutants of key genes which can set seeds in the BR biosynthesis or signaling pathways. To observe if the mutants referred in the context can be used as the tool for genetic screening, the phenotypes of the matured intermediate alleles, such as plant height, rosette width, silique length, and the average seed numbers in each silique were further investigated (Figures 7A–F). Although the weak alleles are all shorter than the wild-type, *dwf4–96, cpd91*, and *dwf5–7* yielded lots of normal seeds (Figures 7A–F). Even if *dwf4–44* is a weak allele showing less compact rosette leaves than the null mutants of *dwf4*, it did not yield any seeds (Figures 7A–F). The seeds of the null mutant *dwf5–8* are fertile, because DWF5 catalyzes the sterol reduction step but not the only way upstream BR biosynthesis (Choe et al., 2000). To test whether the weak alleles are good for genetic modifier screening, an activation tagging approach with a binary vector *pBASTA-AT2* was employed to screen suppressor from *cpd91*. Several suppressors which can partially rescue the dwarf phenotype of *cpd91* were obtained (Figure S7). These results suggest *dwf4–96, cpd91, dwf5–7*, and *dwf5–8* could be used for genetic modifier screen to discover new components involving BR biosynthesis and catabolism pathways.

## Discussion

In the last few decades, BR signaling pathways have been widely elucidated by biochemical and genetic approaches. Reverse genetics is extensively used to uncover the genes participating in BR signal transduction. Because of the gene redundancy in plant genome, a mutant with loss-of-function of a single gene may not show distinct phenotypes compared to that of wild-type plants. Gain-of-function such as activation-tagging approach is an alternative approach to reveal novel genes regulating BR signal transduction. Some of these encoding genes involving in BR signaling pathway are identified via genetic suppressor screen using the weak alleles of *bri1* in Arabidopsis. For instance, BAK1 was identified as a co-receptor of BRI1 by an activation tagging approach searching for genetic suppressors of an intermediate BRI1 mutant allele, *bri1–5* (Noguchi et al., 1999; Li et al., 2002). BRS1 is a serine carboxypeptidase involved in early events in BRI1 signaling pathway (Li et al., 2001; Zhou and Li, 2005), but little is known, so far, about the mechanisms of how BRS1 regulates BRs. BRI1-LIKE RECEPTOR KINASE 1 (BRL1) was identified playing a redundant role with BRI1 in BR signaling (Zhou et al., 2004). BSU1 was identified as a *bri1–5* suppressor by activation tagging (Mora-García et al., 2004). *bes1-D* was found as a suppressor of a weak allele *bri1–119* through EMS mutagenesis (Yin et al., 2002). BES1 and BRASSINAZOLE-RESISTANCE 1 (BRZ1) are two important transcription factors downstream BR signaling, which have demonstrated to play dual roles in BR signaling and biosynthesis regulation (Wang et al., 2002; Yin et al., 2002). BES1 targets a large number of genes such as MYB30 to positively enhance BR signaling and negatively regulates genes involving BR biosynthesis pathways such as *DWF4* and *CPD* in a feed back loop (Li et al., 2009; Ye et al., 2011; Yu et al., 2011). BRZ1 induces the expression of genes to positively amplify BR signals and directly targets BR biosynthetic genes *CPD, DWF4, ROT3*, and *BR6OX* in a negative feedback manner (He et al., 2005).

It is reported that *CPD* is transcriptionally regulated by BRX, the convergence between BR and auxin in root development (Mouchel et al., 2006). Another evidence shows that CESTA directly regulates the expression of *CPD* by binding to its promoter (Poppenberger et al., 2011). A transcription factor TCP1 directly binds to the promoter of *DWF4* and positively regulates the expression of *DWF4* (Guo et al., 2010). However, the detailed molecular mechanisms of BR biosynthesis and catabolism are not well understood yet. For example, It was recently discovered that *DWF4* is also stimulated by auxin, but the intermediate factors are still unknown (Chung et al., 2011). Besides the regulation mechanisms of *CPD* and *DWF4* by known transcription factors, do any other components directly transcriptionally and translationally regulate *CPD, DWF4, DWF5*, and other BR biosynthetic genes? Any other unknown genes involving regulating BR biosynthesis and catabolism? How does *BEN1* function in BR catabolism pathways? These scientific questions needs to be dissolved by different approaches such as using genetics and biochemistry tools.

Because of the possible instability of some derivative of BRs, it's relatively difficult to fully reveal the mechanisms of BR metabolism solely by chemistry or biochemistry approach (Khripach et al., 1999). Genetic methods should be considered in this case. However, it was impeded in researches on BR metabolism by reverse genetics because of limited intermediate alleles of the key genes in BR biosynthesis pathway. In this study, several weak mutants were identified as good tools for genetic suppressor screen for new components in BR metabolism. First, most of the identified BR intermediate alleles can yield normal seeds, which is a critical guarantee of genetic screen. Second, CPD and DWF4 catalyze key steps of BR biosynthesis. Using activation-tagging genetic suppressor screen for the weak mutants *cpd91* and *dwf4–96*, it is likely to find the genes directly activating or inactivating CPD and DWF4. Third, DWF5 is responsible for the sterol biosynthesis upstream of BR. Suppressor screen for *dwf5* mutants may help us to dissect the crosstalk between BR biosynthesis and other sterol biosynthesis.

In conclusion, except for the known intermediate mutants of BR signaling and biosynthesis pathways, *dwf4–96, dwf4–44, cpd91, dwf5–7* and *dwf5–8* are identified as intermediate alleles in BR biosynthesis pathways. These mutants might provide a useful tool for genetic suppressor screen to find out more new components in BR metabolism in the near future.

## Author contributions

JD designed the experiment. JD, BZ, XS, MS, DZ and SZ performed the experiment. JD, BZ, XS, and WY analyzed the data. JD wrote the manuscript.

## Funding

The work was supported by National Natural Science Foundation of China grants 31671445, 31401308, and 31371555, China Postdoctoral Science Foundation Grants 2011M501491 and 2012T50830.

### Conflict of interest statement

The authors declare that the research was conducted in the absence of any commercial or financial relationships that could be construed as a potential conflict of interest.

## Supplementary material

Figure S1 **Semi-quantitative RT-PCR analyses show that *DWF5*, but not several other BR biosynthetic genes, is not detectable in the *dwf5–8* mutant**. Thirty six cycles were used for BR biosynthetic genes, and 19 cycles for *EF1*α for the PCR amplification.

Figure S2 **Genomic DNA fragments are differentially detectable from *dwf5–8* and Col-0 with different primers**. Bands in lane #1 were amplified with primers Bands in lane #1 were amplified with primers DWF5-5UTR F and DWF5-5UTR R, bands in lane #2 were amplified with primers DWF5-Middle F and DWF5-Middle R, and bands in lane #3 were amplified with primers DWF5-3UTR F and DWF5-3UTR R. All PCR products were obtained from a 36-cycle amplification.

Figure S3 **A deletion of –1213 to 376 bp from the start codon of *DWF5* was found in *dwf5–8* mutant**. Green box shows that the deleted genomic fragment in *dwf5-8* mutant.

Figure S4 **qRT-PCR analyses show that expression of *CPD, DWF4*, and *DWF5* was down-regulated or not detectable in corresponding mutants of these genes**. *ROT3* is upregulated whereas *SAUR-AC1* is down-regulated in the in the BR biosynthetic mutants.

Figure S5 **Response of the null and weak mutants to 24-epiBL treatment. (A)** Phenotypes of the mutants with 0, 0.1, 1, 10, 100, and 1000 nM of 24-epiBL treatment. Scale bar represents 1 cm. **(B)** Statistical data of root length of the mutants. Student's *t*-test was used to show significance between the 24-epiBL-untreated and treated root lengths of the seedlings (^***^*P* < 0.001). **(C)** Statistical data of hypocotyl length of the mutants. Student's *t*-test was used to show significance between the 24-epiBL-untreated and treated root lengths of the seedlings (^***^*P* < 0.001).

Figure S6 **Hypocotyl response of the weak and null mutants to BRZ. (A)** Hypocotyl phenotypes of the mutants treated with0, 0.1, and 0.5 μM BRZ. **(B)** Statistical analyses for the hypocotyl length of the null and weak allele mutants. One-tailed *t*-test was used to show significance between the hypocotyl lengths of the BRZ-untreated and treated seedlings (^***^*P* < 0.001). Scale bar represents 1 cm.

Figure S7 **Several genetic modifiers were obtain by activation tagging**. The suppressors of *cpd91* can partially suppress the dwarf phenotype of *cpd91*. Scale bar represents 1 cm.

Table S1 **Primers used in this study**.

## References

[B1] Asami T. Min Y. K. Nagata N. Yamagishi K. Takatsuto S. Fujioka S.. (2000). Characterization of brassinazole, a triazole-type brassinosteroid biosynthesis inhibitor. Plant physiol. 123, 93–100. 10.1104/pp.123.1.93 10806228 PMC58985

[B2] Choe S. Dilkes B. P. Fujioka S. Takatsuto S. Sakurai A. Feldmann K. A. (1998). The DWF4 gene of Arabidopsis encodes a cytochrome P450 that mediates multiple 22α-hydroxylation steps in brassinosteroid biosynthesis. Plant Cell 10, 231–243. 10.1105/tpc.10.2.231 9490746 PMC143988

[B3] Choe S. Dilkes B. P. Gregory B. D. Ross A. S. Yuan H. Noguchi T.. (1999a). The Arabidopsis *dwarf1* mutant is defective in the conversion of 24-methylenecholesterol to campesterol in brassinosteroid biosynthesis. Plant Physiol. 119, 897–908. 10.1104/pp.119.3.897 10069828 PMC32104

[B4] Choe S. Noguchi T. Fujioka S. Takatsuto S. Tissier C. P. Gregory B. D.. (1999b). The Arabidopsis *dwf7/ste1* mutant is defective in the δ^7^ sterol C-5 desaturation step leading to brassinosteroid biosynthesis. Plant Cell 11, 207–221. 9927639 PMC144158

[B5] Choe S. Tanaka A. Noguchi T. Fujioka S. Takatsuto S. Ross A. S. (2000). Lesions in the sterol Δ^7^ reductase gene of Arabidopsis cause dwarfism due to a block in brassinosteroid biosynthesis. Plant J. 21, 431–443. 10.1046/j.1365-313x.2000.00693.x 10758495

[B6] Chory J. Nagpal P. Peto C. A. (1991). Phenotypic and genetic analysis of det2, a new mutant that affects light-regulated seedling development in Arabidopsis. Plant Cell 3, 445–459. 10.1105/tpc.3.5.445 12324600 PMC160013

[B7] Chung Y. Maharjan P. M. Lee O. Fujioka S. Jang S. Kim B.. (2011). Auxin stimulates *DWARF4* expression and brassinosteroid biosynthesis in Arabidopsis. Plant J. 66, 564–578. 10.1111/j.1365-313X.2011.04513.x 21284753

[B8] Clouse S. D. (2002). Arabidopsis mutants reveal multiple roles for sterols in plant development. Plant Cell 14, 1995–2000. 10.1105/tpc.140930 12215500 PMC543216

[B9] Clouse S. D. Sasse J. M. (1998). Brassinosteroids: essential regulators of plant growth and development. Annu. Rev. Plant Physiol. Plant Mol. Biol. 49, 427–451. 10.1146/annurev.arplant.49.1.427 15012241

[B10] Du J. Gao Y. Zhan Y. Zhang S. Wu Y. Xiao Y.. (2016). Nucleocytoplasmic trafficking is essential for BAK1- and BKK1-mediated cell-death control. Plant J. 85, 520–531. 10.1111/tpj.13125 26775605

[B11] Friedrichsen D. M. Joazeiro C. A. Li J. Hunter T. Chory J. (2000). Brassinosteroid-insensitive-1 is a ubiquitously expressed leucine-rich repeat receptor serine/threonine kinase. Plant Physiol. 123, 1247–1256. 10.1104/pp.123.4.1247 10938344 PMC59084

[B12] Fujioka S. Li J. Choi Y. H. Seto H. Takatsuto S. Noguchi T.. (1997). The Arabidopsis deetiolated2 mutant is blocked early in brassinosteroid biosynthesis. Plant Cell 9, 1951–1962. 10.1105/tpc.9.11.1951 9401120 PMC157049

[B13] Gou X. Yin H. He K. Du J. Yi J. Xu S.. (2012). Genetic evidence for an indispensable role of somatic embryogenesis receptor kinases in brassinosteroid signaling. PLoS Genet. 8:e1002452. 10.1371/journal.pgen.1002452 22253607 PMC3257278

[B14] Guo Z. Fujioka S. Blancaflor E. B. Miao S. Gou X. Li J. (2010). TCP1 modulates brassinosteroid biosynthesis by regulating the expression of the key biosynthetic gene *DWARF4* in *Arabidopsis thaliana*. Plant Cell 22, 1161–1173. 10.1105/tpc.109.069203 20435901 PMC2879762

[B15] He J.-X. Gendron J. M. Sun Y. Gampala S. S. Gendron N. Sun C. Q.. (2005). BZR1 is a transcriptional repressor with dual roles in brassinosteroid homeostasis and growth responses. Science 307, 1634–1638. 10.1126/science.1107580 15681342 PMC2925132

[B16] He K. Gou X. Yuan T. Lin H. Asami T. Yoshida S.. (2007). BAK1 and BKK1 regulate brassinosteroid-dependent growth and brassinosteroid-independent cell-death pathways. Curr. Biol. 17, 1109–1115. 10.1016/j.cub.2007.05.036 17600708

[B17] Hong Z. Jin H. Tzfira T. Li J. (2008). Multiple mechanism–mediated retention of a defective brassinosteroid receptor in the endoplasmic reticulum of Arabidopsis. Plant Cell 20, 3418–3429. 10.1105/tpc.108.061879 19060110 PMC2630446

[B18] Jin H. Yan Z. Nam K. H. Li J. (2007). Allele-specific suppression of a defective brassinosteroid receptor reveals a physiological role of UGGT in ER quality control. Mol. Cell 26, 821–830. 10.1016/j.molcel.2007.05.015 17588517 PMC1948852

[B19] Kang B. Wang H. Nam K. H. Li J. Li J. (2010). Activation-tagged suppressors of a weak brassinosteroid receptor mutant. Mol. Plant 3, 260–268. 10.1093/mp/ssp099 19995721 PMC2807927

[B20] Khripach V. A. Zhabinskii V. N. de Groot A. E. (1999). Brassinosteroids: A New Class of Plant Hormones. San Diego, CA: Academic Press.

[B21] Kim G.-T. Fujioka S. Kozuka T. Tax F. E. Takatsuto S. Yoshida S.. (2005). CYP90C1 and CYP90D1 are involved in different steps in the brassinosteroid biosynthesis pathway in *Arabidopsis thaliana*. Plant J. 41, 710–721. 10.1111/j.1365-313X.2004.02330.x 15703058

[B22] Kim G.-T. Tsukaya H. Uchimiya H. (1998). The *ROTUNDIFOLIA3* gene of *Arabidopsis thaliana* encodes a new member of the cytochrome P-450 family that is required for the regulated polar elongation of leaf cells. Genes Dev. 12, 2381–2391. 10.1101/gad.12.15.2381 9694802 PMC317051

[B23] Li J. Chory J. (1997). A putative leucine-rich repeat receptor kinase involved in brassinosteroid signal transduction. Cell 90, 929–938. 10.1016/S0092-8674(00)80357-8 9298904

[B24] Li J. Lease K. A. Tax F. E. Walker J. C. (2001). BRS1, a serine carboxypeptidase, regulates BRI1 signaling in *Arabidopsis thaliana*. Proc. Natl. Acad. Sci. U.S.A. 98, 5916–5921. 10.1073/pnas.091065998 11320207 PMC33313

[B25] Li J. Wen J. Lease K. A. Doke J. T. Tax F. E. Walker J. C. (2002). BAK1, an Arabidopsis LRR receptor-like protein kinase, interacts with BRI1 and modulates brassinosteroid signaling. Cell 110, 213–222. 10.1016/S0092-8674(02)00812-7 12150929

[B26] Li L. Yu X. Thompson A. Guo M. Yoshida S. Asami T.. (2009). Arabidopsis MYB30 is a direct target of BES1 and cooperates with BES1 to regulate brassinosteroid-induced gene expression. Plant J. 58, 275–286. 10.1111/j.1365-313X.2008.03778.x 19170933 PMC2814797

[B27] Mora-García S. Vert G. Yin Y. Caño-Delgado A. Cheong H. Chory J. (2004). Nuclear protein phosphatases with Kelch-repeat domains modulate the response to brassinosteroids in Arabidopsis. Genes Dev. 18, 448–460. 10.1101/gad.1174204 14977918 PMC359398

[B28] Mouchel C. F. Osmont K. S. Hardtke C. S. (2006). BRX mediates feedback between brassinosteroid levels and auxin signalling in root growth. Nature 443, 458–461. 10.1038/nature05130 17006513

[B29] Neff M. M. Nguyen S. M. Malancharuvil E. J. Fujioka S. Noguchi T. Seto H.. (1999). BAS1: a gene regulating brassinosteroid levels and light responsiveness in Arabidopsis. Proc. Natl. Acad. Sci. U.S.A. 96, 15316–15323. 10.1073/pnas.96.26.15316 10611382 PMC24817

[B30] Noguchi T. Fujioka S. Choe S. Takatsuto S. Yoshida S. Yuan H.. (1999). Brassinosteroid-insensitive dwarf mutants of Arabidopsis accumulate brassinosteroids. Plant Physiol. 121, 743–752. 10.1104/pp.121.3.743 10557222 PMC59436

[B31] Ohnishi T. Godza B. Watanabe B. Fujioka S. Hategan L. Ide K.. (2012). CYP90A1/CPD, a brassinosteroid biosynthetic cytochrome P450 of Arabidopsis, catalyzes C-3 oxidation. J. Biol. Chem. 287, 31551–31560. 10.1074/jbc.M112.392720 22822057 PMC3438987

[B32] Poppenberger B. Rozhon W. Khan M. Husar S. Adam G. Luschnig C.. (2011). CESTA, a positive regulator of brassinosteroid biosynthesis. EMBO J. 30, 1149–1161. 10.1038/emboj.2011.35 21336258 PMC3061039

[B33] Ren C. Han C. Peng W. Huang Y. Peng Z. Xiong X.. (2009). A leaky mutation in DWARF4 reveals an antagonistic role of brassinosteroid in the inhibition of root growth by jasmonate in Arabidopsis. Plant Physiol. 151, 1412–1420. 10.1104/pp.109.140202 19741050 PMC2773060

[B34] Roh H. Jeong C. W. Fujioka S. Kim Y. K. Lee S. Ahn J. H.. (2012). Genetic evidence for the reduction of brassinosteroid levels by a BAHD acyltransferase-like protein in Arabidopsis. Plant Physiol. 159, 696–709. 10.1104/pp.112.197202 22544867 PMC3375935

[B35] Schneider K. Breuer C. Kawamura A. Jikumaru Y. Hanada A. Fujioka S.. (2012). Arabidopsis PIZZA has the capacity to acylate brassinosteroids. PLoS ONE 7:e46805. 10.1371/journal.pone.0046805 23071642 PMC3465265

[B36] Shang Y. Lee M. M. Li J. Nam K. H. (2011). Characterization of *cp3* reveals a new *bri1* allele, *bri1-120*, and the importance of the LRR domain of BRI1 mediating BR signaling. BMC Plant Biol. 11:8. 10.1186/1471-2229-11-8 21219661 PMC3024917

[B37] Shimada Y. Goda H. Nakamura A. Takatsuto S. Fujioka S. Yoshida S. (2003). Organ-specific expression of brassinosteroid-biosynthetic genes and distribution of endogenous brassinosteroids in Arabidopsis. Plant Physiol. 131, 287–297. 10.1104/pp.013029 12529536 PMC166808

[B38] Turk E. M. Fujioka S. Seto H. Shimada Y. Takatsuto S. Yoshida S.. (2003). CYP72B1 inactivates brassinosteroid hormones: an intersection between photomorphogenesis and plant steroid signal transduction. Plant Physiol. 133, 1643–1653. 10.1104/pp.103.030882 14605216 PMC300720

[B39] Wang H. Nagegowda D. A. Rawat R. Bouvier-Navé P. Guo D. Bach T. J.. (2012). Overexpression of *Brassica juncea* wild-type and mutant HMG-CoA synthase 1 in Arabidopsis up-regulates genes in sterol biosynthesis and enhances sterol production and stress tolerance. Plant Biotechnol. J. 10, 31–42. 10.1111/j.1467-7652.2011.00631.x 21645203

[B40] Wang H. Zhu Y. Fujioka S. Asami T. Li J. Li J. (2009). Regulation of Arabidopsis brassinosteroid signaling by atypical basic helix-loop-helix proteins. Plant Cell 21, 3781–3791. 10.1105/tpc.109.072504 20023194 PMC2814491

[B41] Wang X. Goshe M. B. Soderblom E. J. Phinney B. S. Kuchar J. A. Li J.. (2005). Identification and functional analysis of *in vivo* phosphorylation sites of the Arabidopsis BRASSINOSTEROID-INSENSITIVE1 receptor kinase. Plant Cell 17, 1685–1703. 10.1105/tpc.105.031393 15894717 PMC1143070

[B42] Wang Z. Y. Nakano T. Gendron J. He J. Chen M. Vafeados D.. (2002). Nuclear-localized BZR1 mediates brassinosteroid-induced growth and feedback suppression of brassinosteroid biosynthesis. Dev. Cell 2, 505–513. 10.1016/S1534-5807(02)00153-3 11970900

[B43] Xu W. Huang J. Li B. Li J. Wang Y. (2008). Is kinase activity essential for biological functions of BRI1? Cell Res. 18, 472–478. 10.1038/cr.2008.36 18332904

[B44] Ye H. Li L. Yin Y. (2011). Recent advances in the regulation of brassinosteroid signaling and biosynthesis pathways. J. Integr. Plant Biol. 53, 455–468. 10.1111/j.1744-7909.2011.01046.x 21554539

[B45] Yin Y. Wang Z.-Y. Mora-Garcia S. Li J. Yoshida S. Asami T.. (2002). BES1 accumulates in the nucleus in response to brassinosteroids to regulate gene expression and promote stem elongation. Cell 109, 181–191. 10.1016/S0092-8674(02)00721-3 12007405

[B46] Yu X. Li L. Zola J. Aluru M. Ye H. Foudree A. (2011). A brassinosteroid transcriptional network revealed by genome-wide identification of BES1 target genes in *Arabidopsis thaliana*. Plant J. 65, 634–646. 10.1111/j.1365-313X.2010.04449.x 21214652

[B47] Yuan T. Fujioka S. Takatsuto S. Matsumoto S. Gou X. He K.. (2007). BEN1, a gene encoding a dihydroflavonol 4-reductase (DFR)-like protein, regulates the levels of brassinosteroids in *Arabidopsis thaliana*. Plant J. 51, 220–233. 10.1111/j.1365-313X.2007.03129.x 17521414

[B48] Zhao B. Li J. (2012). Regulation of brassinosteroid biosynthesis and inactivation. J. Integr. Plant Biol. 54, 746–759. 10.1111/j.1744-7909.2012.01168.x 22963251

[B49] Zhou A. Li J. (2005). Arabidopsis BRS1 Is a secreted and active serine carboxypeptidase. J. Biol. Chem. 280, 35554–35561. 10.1074/jbc.M503299200 16123046

[B50] Zhou A. Wang H. Walker J. C. Li J. (2004). BRL1, a leucine-rich repeat receptor-like protein kinase, is functionally redundant with BRI1 in regulating Arabidopsis brassinosteroid signaling. Plant J. 40, 399–409. 10.1111/j.1365-313X.2004.02214.x 15469497

[B51] Zhu W. Wang H. Fujioka S. Zhou T. Tian H. Tian W.. (2013). Homeostasis of brassinosteroids regulated by DRL1, a putative acyltransferase in Arabidopsis. Mol. Plant 6, 546–558. 10.1093/mp/sss144 23204503

