# Supplementary figures and images for: Identification and Characterization of Multiple Intermediate Alleles of the Key Genes Regulating Brassinosteroid Biosynthesis Pathways
Source: Front Plant Sci. 2017 Jan 16;7:1893. doi: 10.3389/fpls.2016.01893 (PMC5238361; doi:10.3389/fpls.2016.01893)

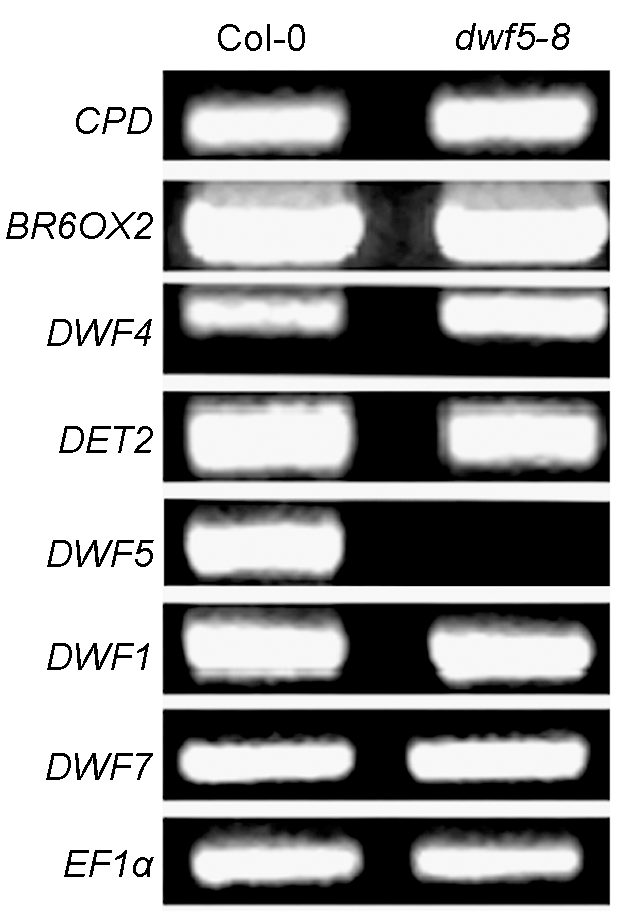

Supplement: Figure S1 — Semi-quantitative RT-PCR analyses show that DWF5, but not several other BR biosynthetic genes, is not detectable in the dwf5–8 mutant. Thirty six cycles were used for BR biosynthetic genes, and 19 cycles for EF1α for the PCR amplification. [file Image1.TIF]

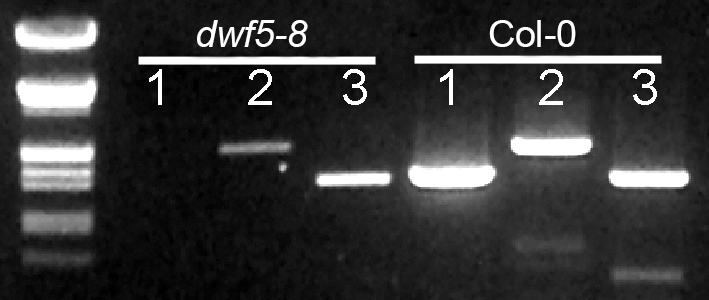

Supplement: Figure S2 — Genomic DNA fragments are differentially detectable from dwf5–8 and Col-0 with different primers. Bands in lane #1 were amplified with primers Bands in lane #1 were amplified with primers DWF5-5UTR F and DWF5-5UTR R, bands in lane #2 were amplified with primers DWF5-Middle F and DWF5-Middle R, and bands in lane #3 were amplified with primers DWF5-3UTR F and DWF5-3UTR R. All PCR products were obtained from a 36-cycle amplification. [file Image2.TIF]

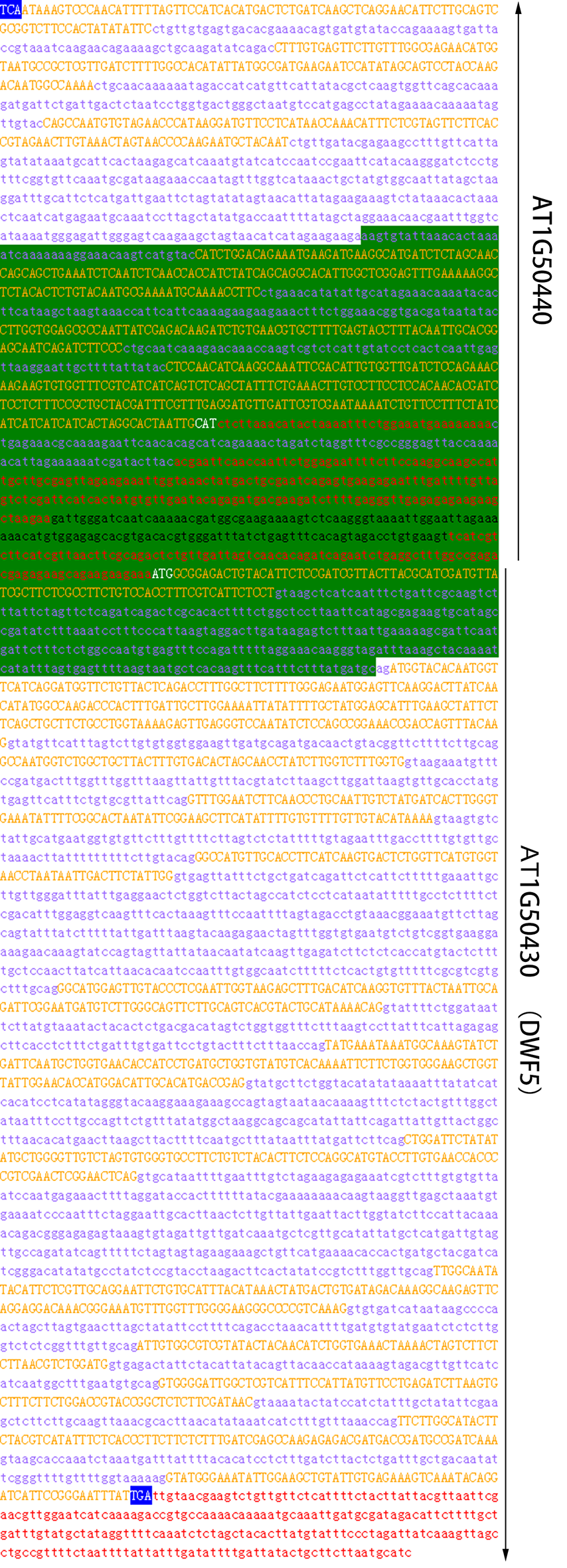

Supplement: Figure S3 — A deletion of –1213 to 376 bp from the start codon of DWF5 was found in dwf5–8 mutant. Green box shows that the deleted genomic fragment in dwf5-8 mutant. [file Image3.TIF]

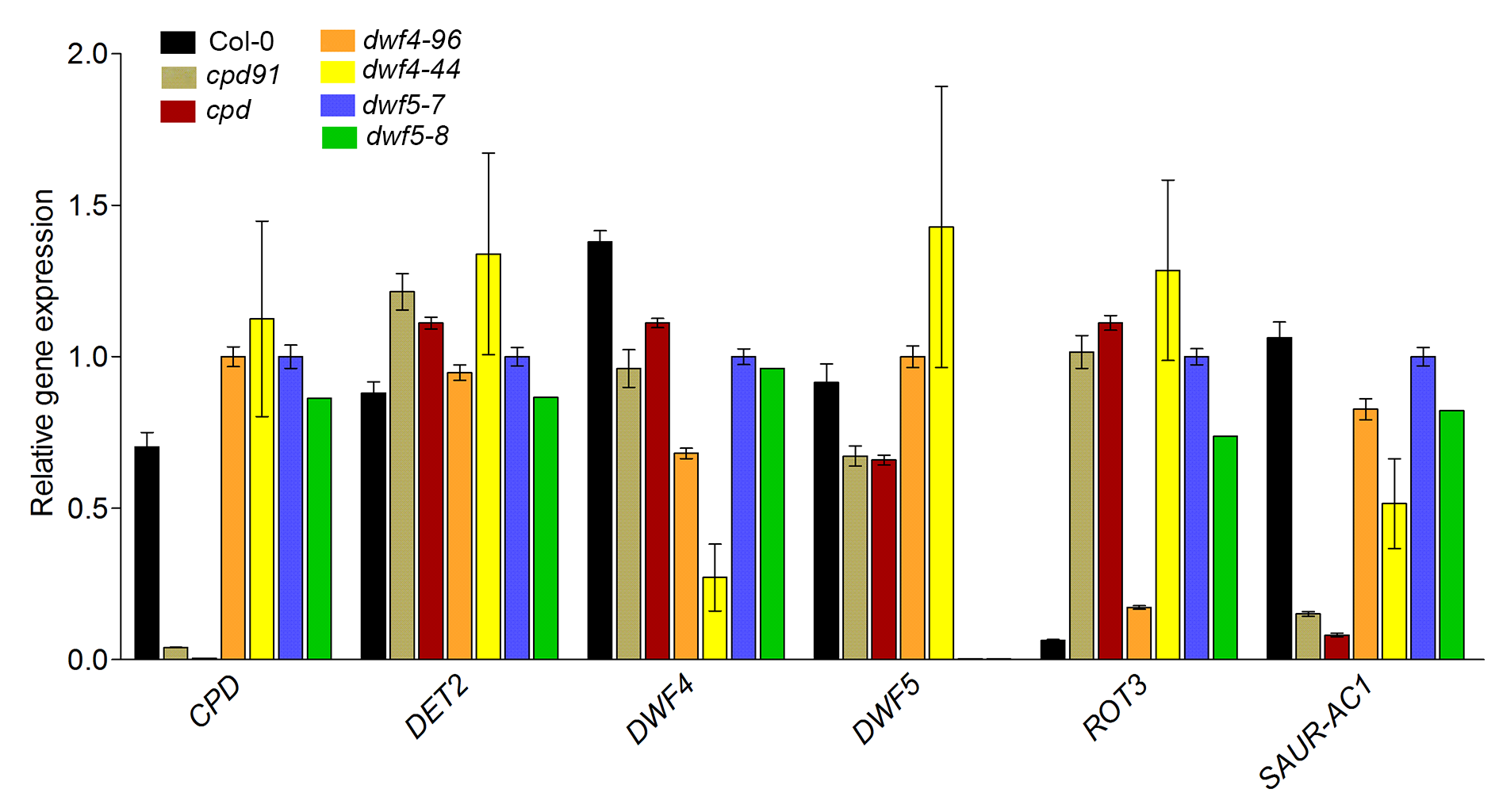

Supplement: Figure S4 — qRT-PCR analyses show that expression of CPD, DWF4, and DWF5 was down-regulated or not detectable in corresponding mutants of these genes. ROT3 is upregulated whereas SAUR-AC1 is down-regulated in the in the BR biosynthetic mutants. [file Image4.TIF]

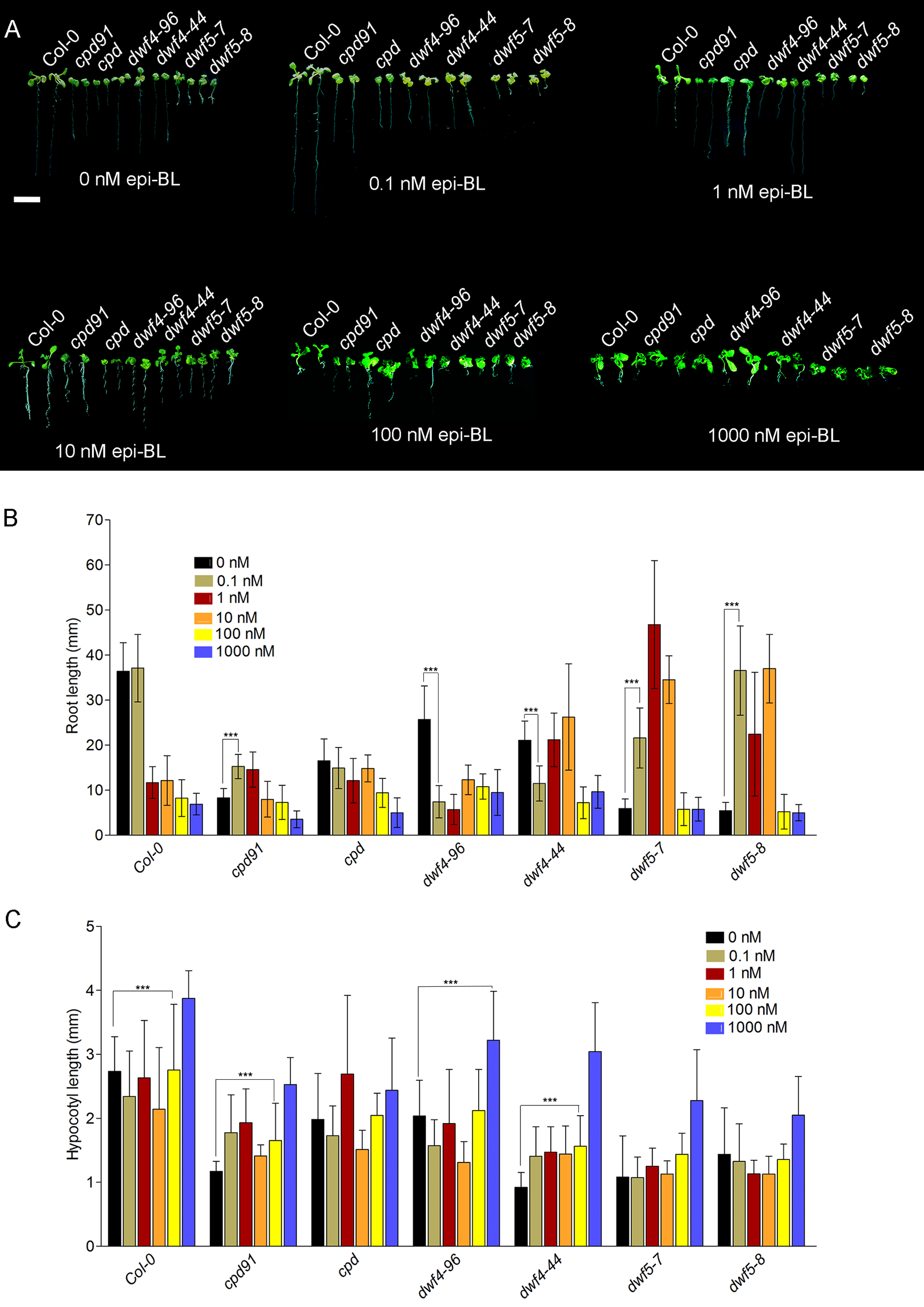

Supplement: Figure S5 — Response of the null and weak mutants to 24-epiBL treatment. (A) Phenotypes of the mutants with 0, 0.1, 1, 10, 100, and 1000 nM of 24-epiBL treatment. Scale bar represents 1 cm. (B) Statistical data of root length of the mutants. Student's t-test was used to show significance between the 24-epiBL-untreated and treated root lengths of the seedlings (***P < 0.001). (C) Statistical data of hypocotyl length of the mutants. Student's t-test was used to show significance between the 24-epiBL-untreated and treated root lengths of the seedlings (***P < 0.001). [file Image5.TIF]

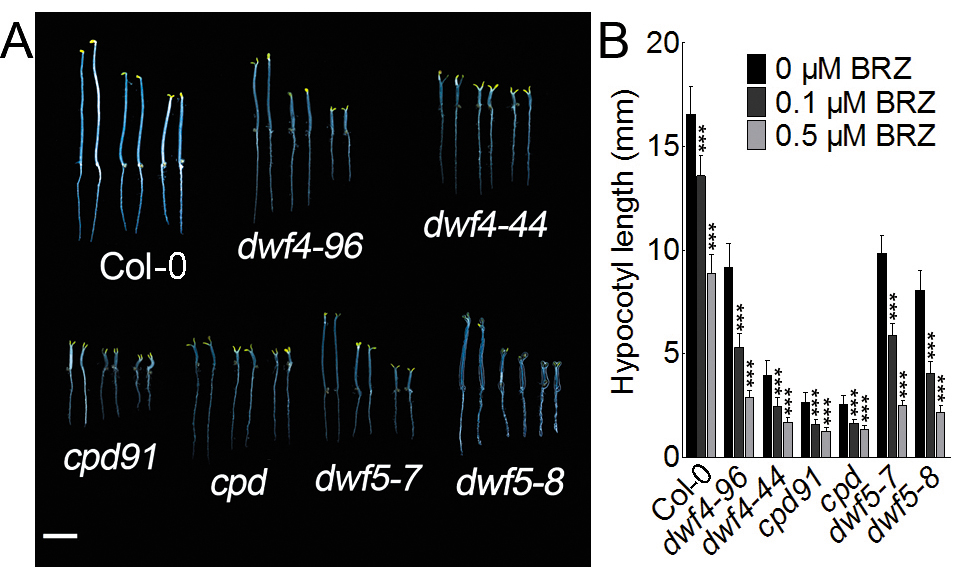

Supplement: Figure S6 — Hypocotyl response of the weak and null mutants to BRZ. (A) Hypocotyl phenotypes of the mutants treated with0, 0.1, and 0.5 μM BRZ. (B) Statistical analyses for the hypocotyl length of the null and weak allele mutants. One-tailed t-test was used to show significance between the hypocotyl lengths of the BRZ-untreated and treated seedlings (***P < 0.001). Scale bar represents 1 cm. [file Image6.TIF]

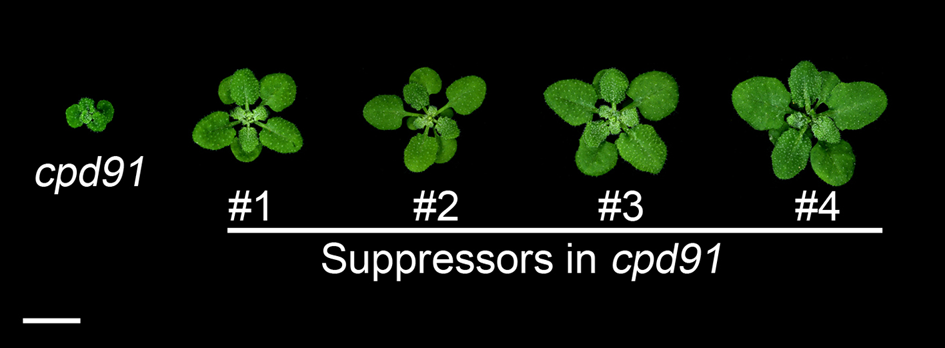

Supplement: Figure S7 — Several genetic modifiers were obtain by activation tagging. The suppressors of cpd91 can partially suppress the dwarf phenotype of cpd91. Scale bar represents 1 cm. [file Image7.TIF]
